# Case of Sloughing Ulceration of the Penis Cured by Vapour-Bathing

**Published:** 1830-10-01

**Authors:** 


### SLOUGHING ULCERATION OF THE PENIS BENEFITTED IN A REMARKABLE MANNER, BY VAPOUR-BATHING.

THE following is an abstract from a paper which we believe was read before the Dublin Association, by Dr. H. C. Field.

A gentleman, aged 36 years, became Dr. Field's patient on the 1st *September*, 1829, for an extensive excoriation around the edge of the prepuce, the result of an imprudent connexion three months previously. The ulceration was daily extending—the penis was swelled, red, shining, and evidently in a state of intense inflammation. The ulceration itself had a dark livid aspect—and a purulent discharge issued from under the prepuce. The patient refused to have the foreskin slit up, there being a natural phymosis, and therefore aperients, poultices, and tranquillity were enjoined. *Sept. 2d.* Great pain extending to the testes—ulceration livid and spreading in a deep circle round the prepuce—febrile excitement. Venesectio ad ℥xx—saline purgatives—poultices—bolus of antimony and opium at bed-time. *5th.* Relieved by the bleeding—but the tumefaction and livid inflammation had extended over the entire penis, which was exquisitely painful. A great portion of the prepuce was now destroyed by the ulceration, and rendered visible a deep foul ulcer of the glans penis. Leeches—fomentations—poultices. The anodyne antimonial at night. *8th Sept.* The swelling is reduced, and the pain diminished; but the ulcer has extended, and has a dark gangrenous appearance, with ragged irregular edges. Almost the whole of the prepuce is gone. The whole of the penis is livid and extremely painful. Around the edge of the ulcer there is a vivid redness—next to this a purplish circle, which loses itself in the dark color of the penis. Leeches

—a mercurial purge. 11*th Sept.* The ulceration is still extending. The nitric acid was applied to the ulcer, and then lint saturated with a strong solution of opium; over all, a fermenting poultice. A grain of opium and 15 drops of nitrous acid to be taken thrice a day. 15*th.* Part of the eschar formed by the acid, and part of the dark gangrenous sloughs came away with each poultice; but the parts beneath were in a state of complete mortification—the integument of the penis was sloughing more rapidly than the deep-seated parts. The patient was in an unhappy nervous state, with distressing palpitation and nervous irritability. Camphor mixture with Hoffman's liquor, 6tis horis—also a drachm of cinchona thrice a day, with the nitrous acid as before. The dressings to be repeated. 18*th.* Dr. Colles was called in consultation. The sloughing was steadily advancing—the pain in the last night had been great—the body of the penis swelled and livid. The same remedies were recommended to be pursued, with the addition of balsam of Peru and carrot poultice. 22*d Sept.* The glans penis was now gone, leaving a surface and pendulous shreds, both gangrenous. A probe sunk deep into a disorganized structure. The patient was dejected—had a small weak pulse, dark-colored tongue, &c. 25*th.* The mortification was now in possession of the corpora cavernosa penis, and rapidly extending towards the pubes. As a last resource the patient was advised to hang the penis, for half an hour at a time, over the steam of hot water, in a metal vessel, with flannel enveloping both the parts and the utensil, in order to retain as much as possible the vapour. The medicines continued, with the exception of the nitrous acid. 27*th Sept.* He was so much relieved by the vapour-bathing, that he slept four hours in the night of the 25th. He therefore applied the vapour six hours daily from that period, as hot as he could possibly bear it. He has had no violent pain since the commencement of the steaming. The sores soon began to assume a more healthy aspect—a granulating point appeared on the left corpus cavernosum—the countenance cleared up—and the inflammation and tumefaction soon disappeared. Quinine was now substituted for the bark. With the exception of a profuse hæmorrhage, which occured on the 3d of October, nothing obstructed the progress towards convalescence, till the 10th of October, when it was found that the granulations had nearly closed the orifice of the urethra, and required the use of the bougie. The irritation produced by this instrument caused some inflammation of the livid kind to return and threaten the patient. This, however, was speedily removed by the vapour-bath, and on the 20th of October, the whole was cicatrized.

---

